# Practitioner-related factors that may influence knowledge and confidence of TDI management in children; a scoping review

**DOI:** 10.1007/s40368-026-01162-6

**Published:** 2026-01-13

**Authors:** R. Eddery, R. Leith

**Affiliations:** Division of Public and Child Dental Health, Dublin Dental University Hospital, Dublin, Ireland

**Keywords:** Traumatic dental injuries, General dental practitioners, Knowledge, Confidence

## Abstract

**Purpose:**

Traumatic dental injuries (TDIs) are the fifth most common chronic disease/injury globally, with 90% of TDIs occurring before the age of 20 years. Initial management of TDIs can influence their prognosis, yet the literature reports a low to moderate knowledge level among practitioners and a reluctance to manage TDIs. The aim of this study was to examine the factors related to general practitioner knowledge and confidence in management of TDIs in children.

**Methods:**

A search strategy was conducted on Pubmed and Embase, with searches limited to English language. Five concepts were addressed using a combination of Emtree/MeSH terms and free-text keywords.

**Results:**

Twenty-three relevant studies were identified. Lack of knowledge and confidence was highlighted worldwide and influenced by practitioner-related factors. Generally, knowledge levels decreased as years since graduation increased. Lack of exposure to TDIs and low self-assessment of knowledge also negatively impacted knowledge. Confidence was lowest among recently qualified practitioners and declined as injury complexity increased. Lack of awareness of recent IADT guidelines negatively affected both knowledge and confidence.

**Conclusion:**

Lack of GDP knowledge of TDI management in children can be associated with being longer qualified, lacking exposure to TDIs, and having a poor self-evaluated knowledge. GDPs who are longer qualified tend to have greater confidence in managing TDIs. Increasing awareness of IADT guidelines is essential for all practitioners. Traumatology mobile applications featuring gamification could be used to engage and promote confidence of newly qualified dentists.

## Introduction

Traumatic dental injuries (TDIs) are the second most common oral disease, after dental caries (Petti et al. 2018). This condition is estimated to be the fifth most prevalent among chronic diseases and injuries worldwide, with a 14.8% and 23.8% incidence in the permanent and primary dentition, respectively (Petti et al. 2018). The highest prevalence of TDIs have been reported in the Americas (Petti et al. 2018; Azami-Aghdash et al. 2015) and occur most commonly in children (Petti et al. 2018; Lam 2016), predominately caused by falls in younger children and contact sports in adolescents (Lam 2016). Maxillary incisors are most frequently affected (Lam 2016; Nagarajappa et al. 2020) with one in ten children in the United Kingdom (UK) sustaining injury to their permanent incisors (Health and Social Care Information Centre 2015). The most common injury to the permanent dentition is enamel fracture, followed by uncomplicated crown fracture (UCF), while subluxation is most prevalent within the primary dentition (Zaleckiene et al. 2014). Timely and appropriate initial management of TDIs is crucial to maximize healing outcomes.

General dental practitioners (GDPs) are at the frontline when managing TDIs for children. Studies from the UK have shown that most children attend their GDP following dental trauma (Maguire et al. 2000; Stewart and Mackie 2004).Therefore, it is reasonable to expect GDPs to be competent in TDI management. Concerningly, these studies also highlighted large numbers of referrals to trauma clinics, with over 40% of children receiving no emergency treatment before referral (Maguire et al. 2000) and over half receiving inadequate treatment (Stewart and Mackie 2004). Reasons for referral included lack of GDP knowledge and confidence in TDI management (Stewart and Mackie 2004).

The International Association of Dental Traumatology (IADT) has published guidelines to support dental practitioners with TDI management (Bourguignon et al. 2020; Fouad et al. 2020; Day et al. 2020). These guidelines were first published in 2001 and most recently updated in 2020, and have been endorsed by the American Association of Endodontists (AAE) and the American Academy of Pediatric Dentistry (AAPD) since 2013. The aim of this study was to evaluate practitioner-related factors that may influence knowledge and confidence of TDI management in children.

## Methods and materials

A scoping review approach was chosen to identify studies examining the factors that influence the knowledge and confidence of GDPs in managing TDIs in children. This approach was chosen to allow us to map the existing literature on this topic. A search strategy was completed on Embase and Pubmed in December 2024. Five concepts were addressed using a combination of Emtree/MeSH terms and free-text keywords.

1. Traumatic dental injuries: "traumatic dental injury" OR "traumatic dental injuries" OR "dental trauma" OR "dental injury" OR "dental traumas" OR "dental injuries".
2. General dental practitioners: "general dentist" OR "general dentists" OR "general dental practitioner" OR "general dental practitioners" OR “dentist” OR “dentists”.
3. Children: “child” OR “children” OR “paediatric patient” OR “paediatric” OR “adolescent” OR “infant”.
4. Knowledge: “knowledge” OR “understanding” OR “understand” OR “knowledgeable” OR “comprehend” OR “comprehension”.
5. Confidence: “confidence” OR “ability” OR “assurance” OR “confident” OR “able” OR “assured”.

Boolean operators (AND/OR) were used in various combinations to increase the specificity of results. Searches were limited to studies in English language. To coincide with the 2012 IADT guidelines update, studies published before 2012 were excluded, as well as studies focusing on knowledge of specialists, students, or regarding management of adult patients (Table 1). Risk of bias assessment was carried out using the JBI critical appraisal checklist for analytical cross-sectional studies (JBI 2020). Each study was assigned a score out of eight based on this checklist, with a score of 3 or less indicating high risk of bias, a score of 4–6 indicating moderate risk of bias, and a score of 7–8 suggesting low risk. One reviewer reviewed all papers and completed risk of bias analysis.

**Table 1:** Results of the included studies

Author	Country	Guidelines	Method of examination	Method of knowledge grading	Determined knowledge	Confidence	Risk of bias
Akhlaghi et al. (2014)	Iran	IADT (2012)	Multiple choice questionnaire on TDI management	Poor 0–4 Moderate 5–8 Good 9–11 Excellent 12–14	Moderate	–	Moderate
Al-Haj Ali et al. (2020)	Saudi Arabia	Unclear	Multiple choice questionnaire on TDI management	1 point per correct answer Categorization undisclosed	Moderate	–	Moderate
Alyasi et al. (2018)	United Arab Emirates	IADT (2012)	Multiple choice questionnaire on TDI management	Inadequate < 6 Adequate ≥ 6	Poor	–	Low
Baginska and Wilczynska-Borawska (2013)	Poland	IADT (2007)	Multiple choice questionnaire on TDI management	1 point per correct answer No categorization, average score 5.76 ± 1.89	Lacked knowledge	–	Moderate
Bani-Hani et al. (2024)	Jordan	–	Questionnaire on experience and self-assessed confidence in pediatric dentistry. Self-assessed confidence reported using Likert scale		–	Low confidence levels in dental trauma	Low
Basir et al. (2018)	Iran	IADT (2012)	Questionnaire on TDI management	Low 10–30 Moderate 30–50 Good 50–70 High > 70	Good overall	–	Low
Buldur and Kaptan (2018)	Turkey	Unclear	Questionnaire on TDI management	Low 0–4 Moderate 5–8 High 9–13 Very high 14–17	Moderate	–	Moderate
Cauwels et al. (2014)	Belgium	IADT (2007)	Multiple choice questionnaire on TDI management	Undisclosed	Insufficient	–	Moderate
Cınar et al. (2013)	Turkey	IADT (2007)	Multiple choice questionnaire on TDI management	Undisclosed	Low	-	Moderate
Cvijic et al. (2024)	Norway	IADT (2020)	Multiple choice questionnaire on TDI management	< 60% considered a low percentage of correct responses	The mean knowledge score was 65.8%	–	Low
Dhaimy et al. (2017)	Morocco	IADT (2012)	Multiple choice questionnaire on TDI management	Undisclosed	Poor	–	Moderate
Duruk and Erel (2020)	Turkey	IADT (2012)	Multiple choice questionnaire on avulsion management	1 point per correct answer Low < 29 Moderate 29 High > 29	Acceptable, but inadequate	–	Low
Hartmann et al. (2018)	Brazil	IADT (2012)	Questionnaire on TDI management	Undisclosed	Moderate	–	Low
Jadav and Abbott (2022)	Australia	IADT (2020)	Questionnaire on TDI management	1 point per correct answer Low 0–3 Acceptable 4–6 Good 7–9 Very good 10–12	Good	–	Low
Kariya et al. (2019)	India	IADT (2012)	Close-ended questionnaire on TDI management	Undisclosed	Inadequate	–	Low
Maloney et al. (2024)	Ireland	IADT (2020)	Multiple choice questionnaire on TDI management	Undisclosed	Good	–	Low
Matoug-Elwerfelli et al. (2022)	Kingdom of Bahrain, Kingdom of Saudi Arabia, Kuwait, Oman, and Qatar	IADT (2020)	Multiple choice questionnaire on TDI management Self-reported confidence on a 5-point Likert scale for various TDIs	Undisclosed	Deficient	Lack of confidence in management of complex injuries and injuries in primary dentition	Low
Ravikumar et al. (2017)	India	AAPD (2013) Consistent with IADT 2012 guidelines	Multiple choice questionnaire on TDI management	Undisclosed	Inconsistent	–	Moderate
Re et al. (2014)	Italy	IADT (2012)	Multiple choice questionnaire on TDI management	Undisclosed	Heterogeneous	–	Low
Skaare et al. (2015)	Norway	IADT (2012)	Multiple choice questionnaire on TDI management, one question allowed text response	Undisclosed	Good	–	Low
Taylor et al. (2021)	England	–	Self-reported confidence on a 5-point Likert scale for various TDIs	–	–	Lack of confidence for complex injuries	Low
Tzanetakis et al. (2021)	Greece	IADT (2020)	Multiple choice questionnaire on TDI management	1 point per correct answer. Inadequate–low 0–3 Acceptable–medium 4–6 Adequate–high 7–8	Acceptable	–	Low
Zaleckienė et al. (2018)	Lithuania	IADT (2012)	Multiple choice questionnaire on TDI management	Undisclosed	Insufficient	–	Low

## Results

**Fig. 1:**
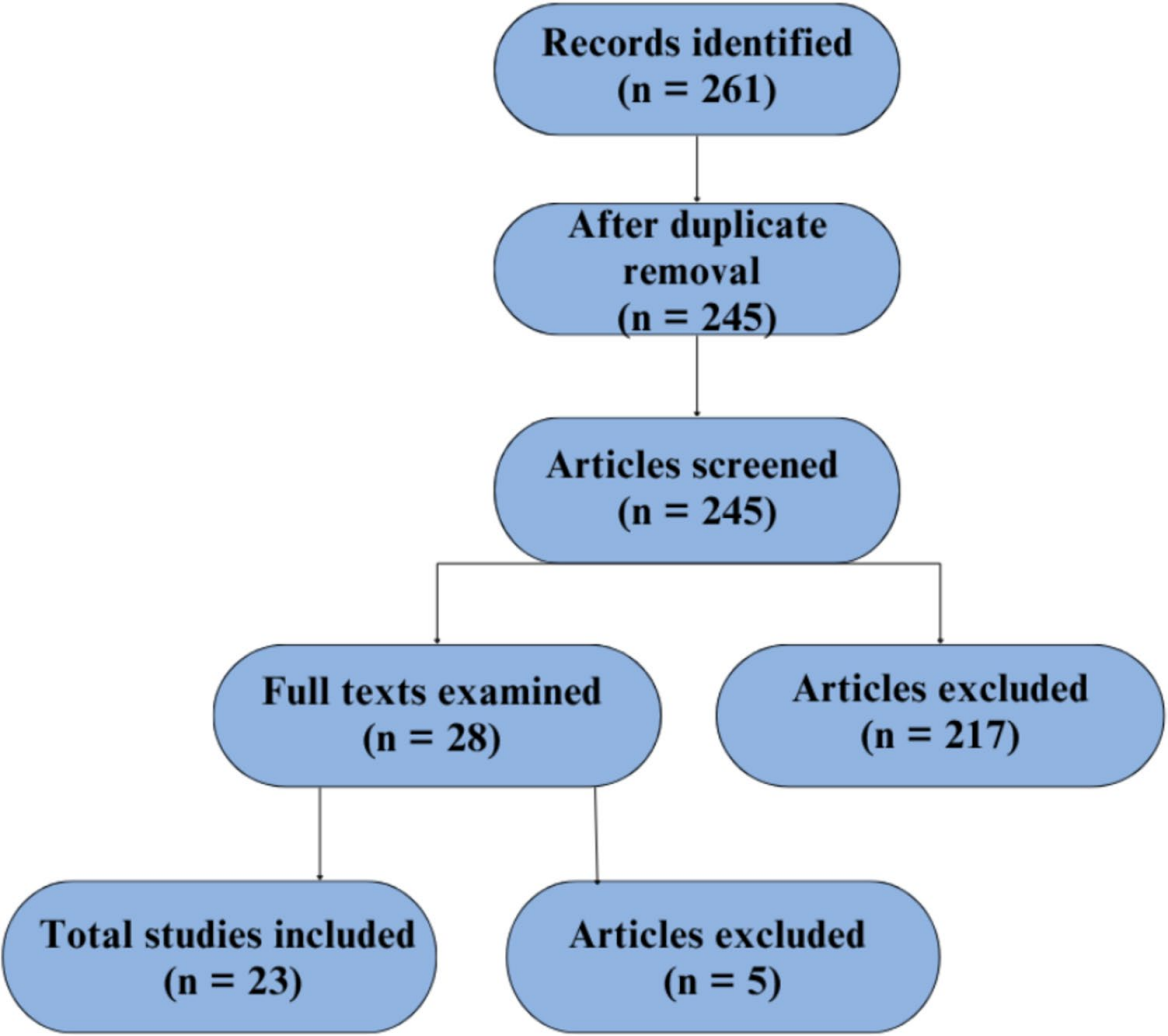
PRISMA flow diagram

**Fig. 2:**
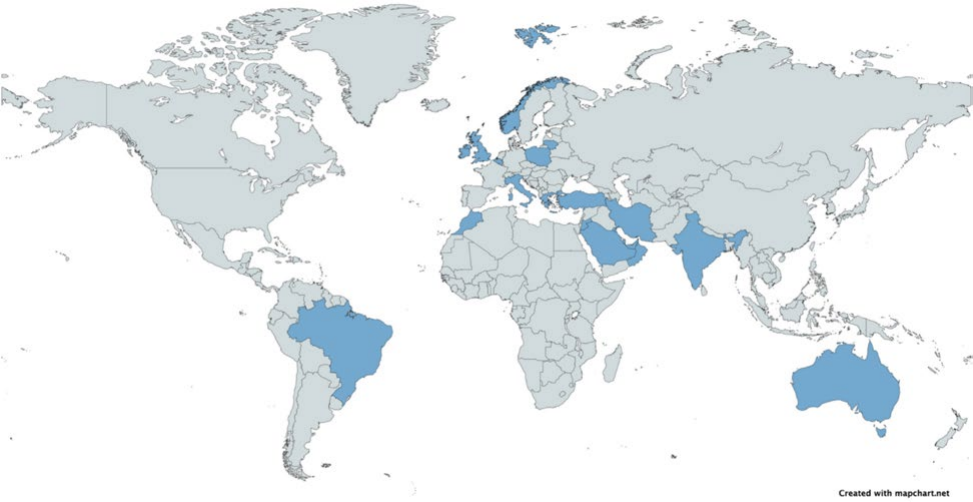
Map displaying the geographical distribution of the studies

Twenty-three studies (Akhlaghi et al. 2014; Al-Haj Ali et al. 2020; Alyasi et al. 2018; Baginska and Wilczynska-Borawska 2013; Bani-Hani et al. 2024; Basir et al. 2018; Buldur and Kaptan 2018; Cauwels et al. 2014; Cınar et al. 2013; Cvijic et al. 2024; Dhaimy et al. 2017; Duruk and Erel 2020; Hartmann et al. 2018; Jadav and Abbott 2022; Kariya et al. 2019; Maloney et al. 2024; Matoug-Elwerfelli et al. 2022; Ravikumar et al. 2017; Re et al. 2014; Skaare et al. 2015; Tzanetakis et al. 2021; Zaleckienė et al. 2018; Taylor et al. 2021) met the inclusion criteria (Fig. 1). As shown in Fig. 2, most studies were conducted in Europe and Asia. Knowledge is reportedly deficient overall, however, there is heterogeneity regarding methods of knowledge level grading and reliability of the studies (Table 1). Only three of the included studies addressed GDP confidence (Bani-Hani et al. 2024; Matoug-Elwerfelli et al. 2022; Taylor et al. 2021) all of which highlighted a lack of confidence in management of TDIs. Eight studies had moderate risk of bias (Akhlaghi et al. 2014; Al-Haj Ali et al. 2020; Baginska and Wilczynska-Borawska 2013; Buldur and Kaptan 2018; Cauwels et al. 2014; Cınar et al. 2013; Dhaimy et al. 2017; Ravikumar et al. 2017) and thirteen studies had low risk of bias (Table 1) (Alyasi et al. 2018; Basir et al. 2018; Cvijic et al. 2024; Duruk and Erel 2020; Hartmann et al. 2018; Jadav and Abbott 2022; Kariya et al. 2019; Maloney et al. 2024; Matoug-Elwerfelli et al. 2022; Re et al. 2014; Skaare et al. 2015; Tzanetakis et al. 2021; Zaleckienė et al. 2018; Bani-Hani et al. 2024). There was heterogeneity between the included studies regarding questionnaire content, knowledge level grading, and risk of bias.

The practitioner-related factors that influenced knowledge of TDI management are summarized in Table 2. Twelve studies found the number of years since qualification of the GDP to be significant, eight found exposure to trauma in practice significant, and a further nine found the practitioner’s self-assessment of their own knowledge significant. The majority of studies indicated that being recently graduated, having experience in TDI management, and being able to accurately self-assess competence improved knowledge of TDI management. Variation was also highlighted with age, gender, and attendance at dental trauma courses.

**Table 2:** Summary of practitioner-related factors that may influence knowledge of TDI management. * Indicates statistically significant results

Author	Years since graduation	Exposure to trauma in practice	Self-assessment of knowledge	Gender	Participation in TDI courses	Age
Akhlaghi et al. (2014)	–	Knowledge increased with exposure.* *p* = 0.004	Self-assessment reflected knowledge.* *p* = 0.01	No significant association	Knowledge increased with participation.* *p* = 0.03	No significant association
Al-Haj Ali et al. (2020)	No significant association	Knowledge increased with exposure.* *p* < 0.001	–	Female GDPs had higher knowledge.* *p* = 0.032	No significant association	No significant association
Alyasi et al. (2018)	–	No significant association	–	No significant association	No significant association	No significant association
Baginska and Wilczynska-Borawska (2013)	GDPs qualified under 10 years showed better knowledge.* *p* = 0.0000	No significant association	No significant association	No significant association	No significant association	–
Basir et al. (2018)	GDPs’ knowledge was highest at 4–7 years, decreasing thereafter.* *p* = 0.000	–	–	Female GDPs had higher knowledge.* *p* = 0.000	No significant association	GDPs under 40 had higher knowledge.* *p* = 0.000
Buldur and Kaptan (2018)	GDPs qualified within 5 years had the highest knowledge.* *p* < 0.05	No significant association	No significant association	No significant association	Knowledge increased with participation.* *p* < 0.05	–
Cauwels et al. (2014)	Knowledge of UCFs improved with years of experience.* *p* = 0.028	Knowledge of deciduous CCFs decreased with exposure.* *p* = 0.011	–	–	–	–
Cınar et al. (2013)	–	–	–	No significant association	–	–
Cvijic et al. (2024)	GDPs qualified within the previous 5 years had significantly higher knowledge than those qualified 6–10 years prior. *p* = 0.016	Knowledge increased with exposure for root fractures *(p* = 0.033), but decreased for avulsions *(p* = 0.0132).*	Self-assessment reflected knowledge regarding intrusion management.* *p* = 0.0344	Male GDPs had higher knowledge of CCFs *(p* = 0.0004) and injuries in immature teeth *(p* = 0.027).*	Knowledge of certain cases increased with participation within the previous 5 years.* *p* = 0.0134	GDPs under 30 years old had higher knowledge.* *p* = 0.014
Dhaimy et al. (2017)	GDPs qualified within 5 years had higher knowledge of CCFs in immature permanent teeth.* *p* = 0.044	Knowledge decreased with exposure for permanent UCFs. GDPs who saw one to two traumas per month were less knowledgeable *(p* = 0.048) than those who saw one to two traumas per year *(p* = 0.024).*	Self-assessment did not reflect knowledge. Longer qualified GDPs assessed their knowledge more highly.* *p* = 0.024	–	Knowledge of UCF management increased with participation.* *p* = 0.006	–
Duruk and Erel (2020)	Dentists graduated within 5 years had the highest knowledge of avulsion management.* *p* < 0.001	Knowledge increased with exposure.* *p* = 0.009	Self-assessment reflected knowledge.* *p* < 0.001	No significant association	–	–
Hartmann et al. (2018)	Dentists with 10–19 years of experience had the highest overall knowledge.* *p* = 0.031	Knowledge increased with exposure.* *p* = 0.000	Self-assessment reflected knowledge.* *p* = 0.000	Female GDPs had higher knowledge.* *p* value not stated	–	–
Jadav and Abbott (2022)	No significant association	Knowledge increased with exposure.* *p* = 0.035	GDPs who assessed their knowledge as ‘very good’ showed higher knowledge than those who assessed their knowledge as ‘good’ (*p* = 0.046), acceptable’(*p* = 0.016), or ‘low’ (*p* = 0.039)	No significant association	–	–
Kariya et al. (2019)	Longer qualified GDPs had higher knowledge of avulsion management* *p* = 0.003 *p* = 0.03	–	–	–	–	–
Maloney et al. (2024)	–	No significant association	Self-assessment reflected knowledge.* *p* < 0.001	No significant association	–	No significant association
Ravikumar et al. (2017)	Longer qualified GDPs had higher knowledge of management of avulsion and extrusion of deciduous teeth.* *p* = 0.02 *p* = 0.04	–	–	–	–	–
Re et al. (2014)	GDPs qualified within 5 years had highest overall knowledge.* *p* = 0.0024	–	–	Female GDPs had higher knowledge of splinting and treatment of UCFs.* *p* = 0.001	No significant association	–
Skaare et al. (2015)	–	–	Self-assessment did not reflect knowledge	–	–	–
Tzanetakis et al. (2021)	GDPs qualified within 5 years had higher knowledge than GDPs qualified over 20 years.* *p* < 0.0001	No significant association	Self-assessment reflected knowledge.* *p* < 0.001	Female GDPs had higher knowledge of avulsion management.* *p* = 0.010	–	GDPs under 45 years old had higher knowledge.* *p* < 0.0001
Zaleckienė et al. (2018)	–	–	Self-assessment reflected knowledge.* *p* = 0.002	–	–	GDPs under 30 years had higher knowledge.* *p* < 0.001

### Years since graduation

Years since graduation was found to significantly influence knowledge in eight studies (Baginska and Wilczynska-Borawska 2013; Basir et al. 2018; Buldur and Kaptan 2018; Cauwels et al. 2014; Cvijic et al. 2024; Dhaimy et al. 2017; Duruk and Erel 2020; Hartmann et al. 2018; Kariya et al. 2019; Ravikumar et al. 2017; Re et al. 2014; Tzanetakis et al. 2021). Most often, it was determined that more recently qualified GDPs had higher knowledge levels (Baginska and Wilczynska-Borawska 2013; Basir et al. 2018; Buldur and Kaptan 2018; Cvijic et al. 2024; Dhaimy et al. 2017; Duruk and Erel 2020; Re et al. 2014). Five studies found that GDPs qualified within 5 years had the highest knowledge levels (Buldur and Kaptan 2018; Cvijic et al. 2024; Dhaimy et al. 2017; Duruk and Erel 2020; Re et al. 2014). Only three studies related more years of experience to greater knowledge, (Hartmann et al. 2018; Cauwels et al. 2014; Kariya et al. 2019), with two of these studies only finding this in relation to specific areas of TDI management, such as managing uncomplicated crown fractures (UCFs) in permanent teeth (Cauwels et al. 2014), splinting times, and tetanus vaccination inquiry (Kariya et al. 2019). Regarding confidence, Matoug-Elwerfelli et al. (2022) reported that GDPs qualified more than 5 years prior showed greater confidence in managing TDIs affecting the permanent dentition than those with less years of experience. Bani-Hani et al. (2024) found that GDPs with 5–10 years of experience were most confident in TDI management.

### Exposure to TDIs

Of the eight studies which found exposure to TDIs significantly influenced knowledge (Akhlaghi et al. 2014; Al-Haj Ali et al. 2020; Cauwels et al. 2014; Cvijic et al. 2024; Dhaimy et al. 2017; Duruk and Erel 2020; Hartmann et al. 2018; Jadav and Abbott 2022), five found knowledge increased with exposure (Akhlaghi et al. 2014; Al-Haj Ali et al. 2020), (Duruk and Erel 2020; Hartmann et al. 2018; Jadav and Abbott 2022). Cvijic et al. (2024) found that with increased exposure, knowledge of root fracture prognosis increased, while knowledge of avulsion management with prolonged extraoral time decreased. Meanwhile, Cauwels et al. (2014) reported GDPs who treated more deciduous complicated crown fractures (CCFs) were less knowledgeable of their management, while Dhaimy et al. (2017) reported similar findings for uncomplicated crown fractures in the permanent dentition. GDPs were found to be less confident when dealing with more complex injuries (Taylor et al. 2021; Matoug-Elwerfelli et al. 2022).

### Self-assessment of knowledge

GDP self-assessment of knowledge was found to accurately reflect knowledge by eight studies (Akhlaghi et al. 2014; Cvijic et al. 2024; Duruk and Erel 2020; Hartmann et al. 2018; Jadav and Abbott 2022; Maloney et al. 2024), while one study found self-assessment to be inaccurate, with more experienced GDPs having higher estimates of their knowledge (Dhaimy et al. 2017).

### Educational courses

Results were inconclusive regarding the influence of attending educational courses, as four studies found an increase in knowledge (Akhlaghi et al. 2014; Buldur and Kaptan 2018; Cvijic et al. 2024; Dhaimy et al. 2017), while three found no increase (Al-Haj Ali et al. 2020; Alyasi et al. 2018; Basir et al. 2018). Only two studies (Basir et al. 2018; Cvijic et al. 2024) asked participants how recently they had completed these courses. No information was gathered in the studies regarding course content and, as there was no baseline knowledge level of GDPs before course attendance, change in knowledge cannot be assessed.

### Age/gender

A small number of studies examined the influence of GDP age on knowledge, with four studies reporting that GDPs below the age of 30–45 years had better knowledge of TDI management (Basir et al. 2018; Cvijic et al. 2024; Tzanetakis et al. 2021; Zaleckienė et al. 2018). Most studies examined the influence of gender on knowledge (Akhlaghi et al. 2014; Al-Haj Ali et al. 2020; Alyasi et al. 2018; Baginska and Wilczynska-Borawska 2013; Basir et al. 2018; Buldur and Kaptan 2018; Cınar et al. 2013; Cvijic et al. 2024; Duruk and Erel 2020; Hartmann et al. 2018; Jadav and Abbott 2022; Maloney et al. 2024; Re et al. 2014; Tzanetakis et al. 2021). Only one study found male GDPs to be more knowledgeable (Cvijic et al. 2024). Five studies found female GDPs to be more knowledgeable (Al-Haj Ali et al. 2020; Basir et al. 2018; Hartmann et al. 2018; Re et al. 2014; Tzanetakis et al. 2021), but females were often overrepresented (Cvijic et al. 2024; Hartmann et al. 2018; Tzanetakis et al. 2021). Overall, eight studies reported no significant difference in male versus female GDP knowledge. Bani-Hani et al. (2024) found male GDPs to be significantly more confident in trauma management.

## Discussion

This review aimed to examine the practitioner-related factors that may influence knowledge and confidence of GDPs in managing TDIs in children. Inadequacies have previously been demonstrated in dentists’ knowledge of emergency dental trauma management (De França et al. 2007; Kostopoulou and Duggal 2005; Yeng and Parashos 2008; Pedrini et al. 2011; Vasanthakumari A 2022; Maloney et al. 2024). Dental trauma management has also been shown to be an area of pediatric dentistry in which GDPs are least confident (Bani-Hani et al. 2024). In general, the results of this review highlighted a lack of both knowledge and confidence.

Our results suggest the possibility of an interesting inverse relationship between knowledge and confidence of TDI management with regard to years of experience. While more recently qualified GDPs have been shown to have the highest knowledge in TDI management (Baginska and Wilczynska-Borawska 2013; Basir et al. 2018; Buldur and Kaptan 2018; Cvijic et al. 2024; Dhaimy et al. 2017; Duruk and Erel 2020; Re et al. 2014), they also show the least confidence (Matoug-Elwerfelli et al. 2022; Bani-Hani et al. 2024). De França et al. (2007) and Kostopoulou and Duggal (2005) also found more recently qualified GDPs had better knowledge in dental trauma management. Newly qualified GDPs should be expected to have the highest knowledge, as they have the most recent education in dental traumatology, likely based on the latest IADT guidelines. They are also more likely to be revising for specialty examinations, as previously suggested by Baginska and Wilczynska-Borawska (2013). Similarly, we can assume that longer qualified GDPs would be more confident from clinical experience, but may not be reviewing the latest guidelines. This highlights the need for GDPs to regularly review the IADT guidelines in practice.

Our results emphasize the positive impact of experience in TDI management on knowledge (Akhlaghi et al. 2014; Al-Haj Ali et al. 2020; Duruk and Erel 2020; Hartmann et al. 2018; Jadav and Abbott 2022). A small number of studies found the level of exposure to TDI cases in practice did not always equate to higher knowledge, again highlighting the importance of regular guideline review. In addition, the finding of greater confidence in GDPs in treating less complex injuries (Taylor et al. 2021; Matoug-Elwerfelli et al. 2022), which tend to occur more frequently, suggests confidence also grows with exposure to dental trauma. The spontaneous nature of TDIs means that dentists lack regular exposure to children’s dental trauma in practice. Zaleckienė et al. (2018) reported that 82.3% of GDPs in Lithuania had treated only single trauma cases, while only 3.3% of GDPs reported providing such treatment regularly. A study in England found 61% of GDPs had never replanted an avulsed tooth in a child (Kenny et al. 2015). However, a considerable number of TDI patients present directly to hospital emergency departments (Huh et al. 2022), rather than to private practice. Many GDPs refer TDI patients to colleagues after emergency treatment (Zaleckienė et al. 2018), meaning they do not evaluate long-term follow-up or assess treatment outcomes. Furthermore, issues with behavior management are common when dealing with pediatric patients (Wu et al. 2021) and this may also prompt referral. Taylor et al. (2021) found inadequate financial remuneration encouraged GDPs to refer TDIs for both short and long-term management. Lack of financial remuneration has previously been highlighted as a barrier to treatment of TDIs in the literature (Yeng and Parashos 2008; Kostopoulou and Duggal 2005). Additionally, increased concern amongst about litigation as a result of TDI mismanagement has been suggested, which may discourage GDPs taking on TDI cases (Taylor et al. 2021).

Accurate self-assessment of knowledge is a vital skill for GDPs, as recognition of low knowledge should encourage revision. While this review most often found self-assessment to accurately reflect knowledge (Akhlaghi et al. 2014; Cvijic et al. 2024; Duruk and Erel 2020; Hartmann et al. 2018; Jadav and Abbott 2022; Maloney et al. 2024), Dhaimy et al. (2017) found that longer-qualified GDPs had the highest confidence in their knowledge, despite recent graduates having better knowledge. This, again, suggests confidence grows with experience while knowledge becomes outdated as new guidelines are published.

The mixed results regarding the impact of continuing profession development (CPD) courses on TDI management can likely be explained by the insufficient data collected by the studies on this subject. Only two studies (Basir et al. 2018; Cvijic et al. 2024) asked participants how recently they had completed these courses, which would affect relevance of the information provided. Basir et al. (2018) found that most participants had last attended a course over 2 years ago, but failed to give participants the option of saying they had never attended a course. No information was gathered in the studies regarding course content, and as the baseline knowledge of GDPs before course attendance was not evaluated, change in knowledge cannot be assessed. Taylor et al. (2021) reported that most GDPs believe continuing education in dental trauma is necessary to ensure appropriate management in practice, but again question the financial return such resources offer. Matoug-Elwerfelli et al. (2022) found attendance at dental trauma courses lead to increased confidence in TDI management, with many GDPs expressing interest in attending such courses. This shows that demand exists amongst GDPs for TDI management courses, but course availability may be scarce.

Most studies failed to ask participants whether they were up to date with the most recent IADT guidelines. Maloney et al. (2024) found that less than half of Irish GDPs in 2024 were up to date with the most recent (2020) iteration of the IADT guidelines, while 30% were completely unaware of the guidelines. Another recent study showed that only 13% of GDPs were familiar with the latest IADT guidelines (Matoug-Elwerfelli et al. 2022). According to Maloney et al. (2024), GDPs who had read the 2020 IADT guidelines had significantly higher knowledge of TDI management than those who had not. Awareness of most recent IADT guidelines has also been associated with greater confidence in management of dental trauma cases amongst GDPs (Matoug-Elwerfelli et al. 2022). Confidence has also been shown to increase with usage of the Dental Trauma Guide (DTG) Matoug-Elwerfelli et al. (2022); however, less than one-third of GDPs have used this subscription based resource (Maloney et al. 2024; Matoug-Elwerfelli et al. 2022; Taylor et al. 2021).

Only three of the included studies examined GDP confidence (Bani-Hani et al. 2024; Taylor et al. 2021; Matoug-Elwerfelli et al. 2022). The subjective nature of confidence makes it more difficult to quantify, as it requires self-assessment by GDPs themselves. In contrast, knowledge can be more objectively assessed through administration of questionnaires which examine different areas of trauma management. This may explain the imbalance between the number of studies examining knowledge compared to confidence.

GDPs should be encouraged to revise the IADT guidelines regularly, and the benefits of the DTG should be recommended, especially to longer qualified GDPs who many not be aware of all resources available. Continued translation of the IADT guidelines and DTG is required to ensure all GDPs have equal access to the most recent knowledge of TDI management in children (Matoug-Elwerfelli et al. 2022). Attention should be focused on creation of further resources to support GDPs in practice. Clinical decision support tools (CDSTs) are algorithms which support healthcare professionals in clinical situations (Huh et al. 2022). Several studies have designed CDSTs for TDI management and proven their benefits in diagnosis and management for students and dental practitioners (Huh et al. 2022; Sahni and Gupta 2022; Machado et al. 2018). Mobile applications provide an ideal platform for such tools. Unfortunately, existing dental trauma applications have been found to be of moderate to low quality (Walia et al. 2024). Endorsement of appropriate applications by the IADT would ensure reliability of the content provided. The ToothSOS app, launched in 2018, is a free service created by the IADT, offering guideline-based trauma algorithms aimed at the general public, and also providing practitioners easy access to relevant guidelines (Tewari et al. 2024). One study found a notable improvement in dental students’ knowledge of TDIs using the patient-directed section of the ToothSOS app (Ozdemir et al. 2025). Likewise, GDPs may develop greater familiarity with guidelines by using this app. Integration of gamification features could be used to further enhance the educational features of such apps and promote continued learning by encouraging user engagement, enjoyability, and motivation (Sardi et al. 2017).

## Conclusion

Several practitioner-related factors influence the knowledge and confidence of GDPs regarding TDI management in children. In general, being longer qualified, having limited exposure to TDIs, and having poor ability to self-evaluate knowledge will mean a GDP is more likely to lack adequate knowledge to manage these patients. GDP confidence is negatively impacted by limited clinical experience and increased complexity of the trauma case. Increasing guideline awareness among GDPs through CPD and integration in mobile applications is recommended.

## Data Availability

No datasets were generated or analysed during the current study.
